# Different phenotypes and factors associated with atopic dermatitis in the young adult Singaporean Chinese population: A cross-sectional study

**DOI:** 10.1016/j.waojou.2018.11.006

**Published:** 2019-01-26

**Authors:** Sri Anusha Matta, Sandrine Blanchet-Rethore, Yang Yie Sio, Bani Kaur Suri, Anand Kumar Andiappan, Ramani Anantharaman, Christophe Piketty, Valerie Bourdes, Fook Tim Chew

**Affiliations:** a Department of Biological Science, National University of Singapore, Singapore; b Nestlé Skin Health–Galderma R&D, Sophia Antipolis, Biot, France; c Singapore Immunology Network (SIgN), Agency for Science Technology and Research (A*STAR), Singapore

**Keywords:** Atopic dermatitis, Allergy, Singaporean Chinese population, Phenotypes, Risk factors, AD, atopic dermatitis, BMI, body mass index, AR, allergic rhinitis, NUS, National University of Singapore, ISAAC, International Study of Asthma and Allergies in Childhood, SPT, skin prick test, HDM, house dust mites

## Abstract

**Background:**

Atopic dermatitis (AD) is a chronic allergic disease typically accompanied by atopy and thus, a tendency to develop allergic diseases such as allergic rhinitis, asthma or food allergies. Currently, individuals with AD are classified into those presenting with AD alone and those presenting with AD along with other allergic diseases (AD+). It is important to identify the various endophenotypes of AD using anthropometric, environmental, socio-economic, and disease history data in order to improve disease management. To characterize the phenotypic differences among Singaporean Chinese individuals with AD alone and AD+, and identify the socioeconomic, lifestyle, and environmental factors associated with these different presentations.

**Methods:**

Based on data collected via a standardized/validated questionnaire, 4604 participants (mean age: 22.1 years) were classified into three groups: 1) AD alone group; 2) AD with other allergic diseases group (AD+); and 3) Control group.

**Results:**

Participants were less sensitized to common inhalant allergens in the AD alone group versus the Control group (67% vs. 72%, respectively; p < 0.05).

High Body Mass Index (i.e., BMI > 23) was associated with the disease and the difference was more pronounced in the AD alone group compared to the AD+ group (Odds Ratio: 1.38; 95% Confidence Interval: 1.4–1.67; p < 0.001). No major differences in habits were observed between the AD alone and AD+ groups.

**Conclusions:**

The two presentations of AD may have different underlying pathogenesis and associated risk factors.

## Background

Atopic dermatitis (AD) is a chronic allergic disease characterized by itchy, inflamed skin, most commonly manifesting at flexural areas.^1^ Typically, AD is accompanied by atopy (i.e., elevated levels of the allergen-specific immunoglobulin E in response to common allergens^2^, ^3^) and thus, a tendency to develop allergic diseases. AD is used as an umbrella term to describe both atopic (extrinsic AD) as well as non-atopic (intrinsic AD) individuals with flexural skin rashes.^4^, ^5^, ^6^

More than 70% of the population in Singapore is atopic.^7^ The prevalence of atopy has been increasing in developing countries, paralleled by a steady increase in obesity. A number of cross-sectional and prospective cohort studies have shown a correlation between atopy and high Body Mass Index (BMI), particularly in individuals suffering from asthma.^6^, ^8^, ^9^, ^10^, ^11^, ^12^ Changes in cytokine and adipokine levels, associated with an increase in body weight, may lead to a decreased immune tolerance to common allergens, thus increasing the susceptibility to atopic conditions.^12^ Studies have shown that adiponectin levels vary among intrinsic and extrinsic AD patients.^13^

Moreover, an increase in the activity of skin proteases along with dysregulation of structural proteins lead to skin barrier dysfunction, allowing easy access to allergens.^14^, ^15^, ^16^

AD may occur at any stage in the life span of an individual; however, it is most commonly reported in early life.^17^ Most individuals diagnosed with AD in their early childhood have a higher risk of developing other atopic conditions such as allergic rhinitis (AR) and asthma in a process termed atopic march.^18^, ^19^, ^20^, ^21^ Mutations in the filaggrin gene have also been shown to be a risk factor for early-onset AD persisting into adulthood.^22^

Although AD is approached as a single disease, it is increasingly recognized that a more tailored approach may be necessary to adequately address the needs of patients. An advanced understanding of individual phenotypic, epidemiological, genetic, and immunological parameters is essential. It is imperative that adequate stratification is performed in epidemiological studies to identify the various endophenotypes of AD using anthropometric, environmental, socio-economic, and disease history data. This approach may further advance our understanding of the condition and lead to improved disease management.^17^ Currently, individuals with AD may be stratified into two groups, namely those who present AD alone and those who present AD along with other allergic diseases.

The objective of this study was to characterize the phenotypic differences, if any, among Singaporean Chinese participants with AD alone and AD with other allergic diseases (vs. healthy participants), and identify the socioeconomic, lifestyle, and environmental factors associated with these different presentations.

## Methods

### Study design

The Singapore Cross-Sectional Asthma and Allergic Diseases Genetic Epidemiology Cohort study was conducted from March 2007 to August 2015 at the National University of Singapore (NUS) in Singapore. This was a cross-sectional study of allergic diseases assessing genetic and environmental factors in recruited volunteers. Since the study was conducted in a university, the population recruited consists mostly of young university students. Participants were recruited through advertisements made via email and posters across the university. Only participants who were above 18 years of age and those not currently taking antihistamines were allowed to take part in the study.

The study size was calculated in accordance with the formula for estimating power in a two-tailed test. In order to use a confidence interval of 95%, a statistical power of 85% and a small effect size (0.2), a total of at least 450 individuals per category were required.^23^

### Data sources/variables

A standardized and validated questionnaire, based on The International Study of Asthma and Allergies in Childhood (ISAAC) questionnaire, was used in this study.^24^ The questions included in the ISAAC questionnaire collected participant-reported information on current (≤12 months) and past (>12 months) symptoms for asthma, AR and AD. In addition, the questionnaire included sections aimed at gathering participant demographic, medical, dietary, lifestyle, and family data. Anthropometric measurements (height and weight) were also surveyed and these data were used to calculate the BMI of participants.

Skin prick tests (SPTs) were conducted by trained personnel to determine the atopy status of participants. SPTs for allergic reactions were performed as described previously.^25^ A wheal larger than 3 mm in diameter was indicative of a positive reaction.

All data (socioeconomic, lifestyle, environmental, and physiological factors), together with the clinical presentations of the participants and SPT results, were analyzed to identify differences between the participant groups as well as individual factors associated with each presentation.

Bias may potentially occur in a hospital setting where only patients with severe AD may be recruited. This bias was avoided by conducting all the cross-sectional studies in an academic setting (university), to capture mild, moderate and severe forms of the disease.

### Participants

Volunteer participants were recruited through multiple recruitment programs across a ten-year period and classified into disease categories based on their responses to the questionnaire. Participants who answered affirmatively to the questions: *“Have you ever had an itchy rash?”* and *“Has this rash affected your flexural areas?”* were identified as suffering from AD. There was no physical examination of presence of any visual flexural rash. However, the research team administered the responses of the participants to the questions in the survey questionnaire. Moreover, participants suffering from asthma and AR were also identified as described previously.^5^, ^25^ Based on this approach, participants were classified into the following three groups:

• AD alone group (presence of symptoms of AD without asthma or AR)
• AD with other allergic diseases group (AD+) (presence of symptoms of AD with either asthma or AR)
• Control group (absence of symptoms of AD).

### Statistical analysis

A chi-square test was used to compare differences between groups. Statistical analysis was performed using the GraphPad Prism 6.0 software (GraphPad Software, Inc., USA). For the regression analysis, the IBM SPSS^®^ 24 (IBM^®^, USA) was used. Binary logistic regression was used with income, years of residence in Singapore, and gender (which were independently found to be significantly associated between the groups) as independent covariates, and the disease status as a dependent variable. Samples with missing data were omitted from the corresponding analyses.

## Results

### Participant disposition

A total of 11,573 participants were recruited during the course of the study. Data analyses were restricted to those of ethnic Chinese origin residing in Singapore, which constituted the vast majority of participants recruited in this study (i.e., N = 10,074). Other ethnicities (N = 1499) were excluded.

The current cohort included 4604 participants, classified into the three aforementioned groups (i.e., AD alone, AD+, and Control). Those who had other allergic diseases and not AD, along with those who had a positive skin prick reaction in the absence of symptoms (N = 5470) were excluded. The cohort (4,604) was divided into the three groups (AD alone, AD+ and Control), including 496, 894, and 3214 participants, respectively. The current cohort had a mean age of 22.1 years (±4.3 years). Participant characteristics are shown in Table 1. Approximately 90% of participants in the AD alone and AD+ groups reported rashes within the previous 12 months (Table 2).

**Table 1:** Demographics and participant characteristics.

	AD alone group (N = 496)	AD with allergic diseases group (N = 894)	Control group (N = 3214)
**Age**	22.34 ± 4.82	22.04 ± 4.27	22.12 ± 4.17
**Gender (Male)**	179 (36.2%)**	375 (42%)	1447 (45.1%)
**Ethnicity (Chinese)**	496 (100%)	894 (100%)	3213 (100%)
**Country of birth (Singapore)**	307 (61.8%)	692 (77.4%)^††^	2163 (67.2%)
**Number of years in Singapore**			
<10	136 (43%)	161 (26.8%)^††††^	669 (37%)
≥10	180 (57%)	441 (73.2%)^††††^	1141 (63%)
**Type of housing**			
HDB (Government housing)	309 (68%)	627 (68%)	588 (69%)
Condominium (Private housing)	84 (19%)	184 (20%)	164 (19%)
Landed	59 (13%)	108 (12%)	105 (12%)
**Number of people in the household**			
≤4	277 (56%)	511 (57.5%)	1805 (57.6%)
>4	219 (44%)	378 (42.5%)	1329 (42.4%)
**Total monthly family income (SGD)**			
<2000	108 (23.1%)	163 (18.6%)^††††^	807 (26.1%)
≥2000	360 (76.9%)	712 (81.4%)^††††^	2285 (73.9%)
**Body Mass Index (kg/m**^**2**^**)**	21.01 ± 2.8	21.12 ± 2.90	20.6 ± 2.78

Values are expressed as the mean ± standard deviation or number (%).

*p < 0.05, **p < 0.01 and ***p < 0.001 and ****p < 0.0001 (AD alone vs. Control).

^†^p < 0.05, ^††^p < 0.01, ^†††^p < 0.001 and^††††^p < 0.0001; (AD+ vs. Control).

**Table 2:** Atopic dermatitis characteristics.

	AD alone group (N = 496)	AD with allergic diseases group (N = 894)	Control group (N = 3214)
**Itchy rash coming and going for 6 months**	496 (100%)****	894 (100%)^††††^	134 (4.2%)
**Had the rash in the past 12 months**	449 (90.7%)****	788 (89%)^††††^	157 (12%)
**Rash affecting flexural areas**	496 (100%)****	894 (100%)^††††^	58 (5%)
**The rash cleared completely in the past 12 months**	317 (65%)	562 (64%)	N/A
**Kept awake at night by the itchy rash**			
Never in the past 12 months	281 (58%)	515 (60%)	N/A
Fewer than one night per week	146 (30%)	274 (32%)	N/A
One or more nights per week	57 (12%)	73 (8%)	N/A
**Suffered from dry skin**	128 (26%)***	292 (33%)^†††^	55 (2%)

Values are expressed as the mean ± standard deviation or number (%).

*p < 0.05, **p < 0.01 and ***p < 0.001 and ****p < 0.0001 (AD alone vs. Control).

^†^p < 0.05, ^††^p < 0.01, ^†††^p < 0.001., ^†††^p < 0.0001 (AD+ vs. Control).

There were no significant differences observed in age, number of individuals residing in the household, and the type of house dwellings between the three groups. However, there was a significant underrepresentation of males in the AD alone group (36.2%) compared to the AD+ (42.0%) and Control (45.1%) groups (p < 0.01). In addition, there was a higher proportion of overseas-born Singaporean Chinese in the AD alone (38.2%) and Control (32.8%) groups compared to the AD+ (22.6%) group (p < 0.01). A significantly higher proportion of participants who had resided in Singapore for more than 10 years was identified in the AD+ group compared to the other two groups (p < 0.0001). Furthermore, higher family income was associated with the AD+ group compared to the other two groups (p < 0.0001).

Analysis of lifestyle factors (i.e., physical activity and smoking status) revealed no significant differences among the AD+, AD alone, and Control groups. Analysis of prescribed medication revealed a higher proportion of participants in the AD+ and AD alone groups who were prescribed antihistamines, topical steroids, and moisturizers compared to the Control group (Supplementary Table 1).

Assessment of food habits did not demonstrate notable differences between groups, apart from a slight increase in the intake of butter observed in the AD alone group and a reduction in the consumption of pulses in the AD+ group (Supplementary Table 2).

Nearly two-thirds (65%) of participants in the AD alone and AD+ groups reported that the rash was acute, resolving completely within the previous 12 months. Of note, significantly more participants in the AD alone and AD+ groups suffered from self-reported dry skin (p < 0.001) (Table 2).

### Sensitization to common inhalant allergens

**Fig. 1:**
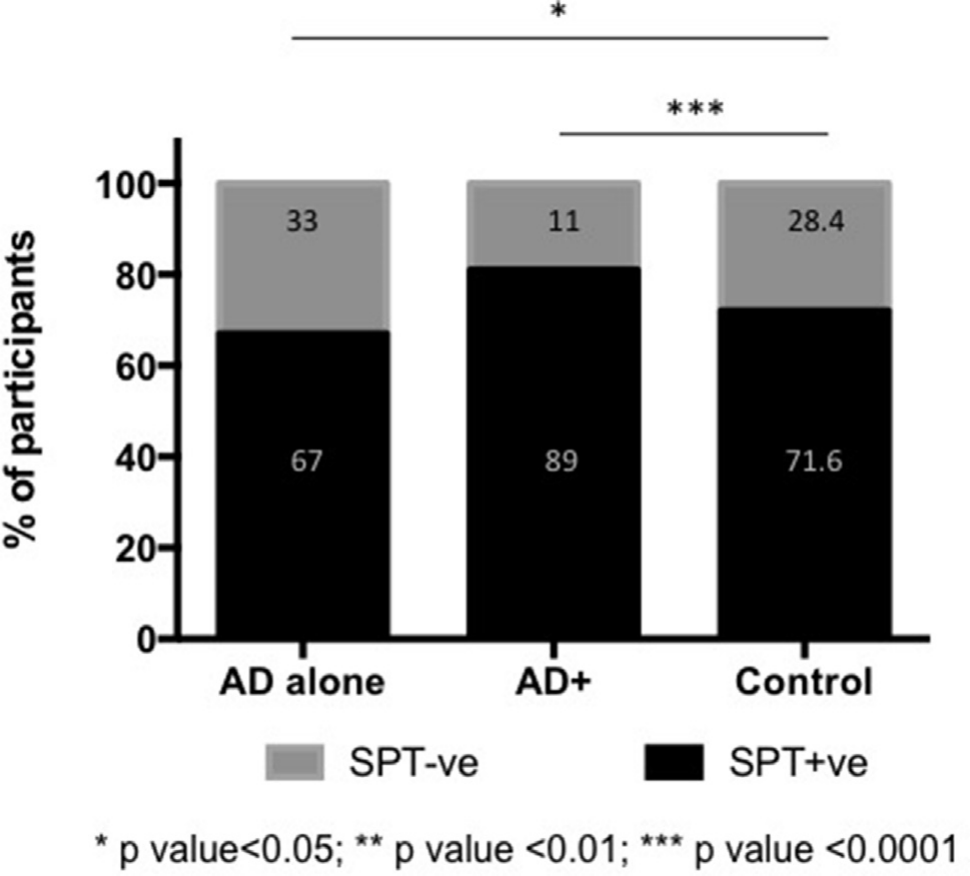
Sensitization to common aeroallergens *p < 0.05; **p < 0.01; ***p < 0.0001.

According to the results of the SPTs, the proportion of participants sensitized to common inhalant allergens in Singapore such as house dust mites (HDM) were 67%, 89%, and 72% in the AD alone, AD+, and Control groups, respectively (Fig. 1). The differences between the two AD groups versus the Control group were statistically significant (AD alone vs. Control [p < 0.05] and AD+ vs. Control [p < 0.0001], respectively).

### AD and BMI

**Fig. 2:**
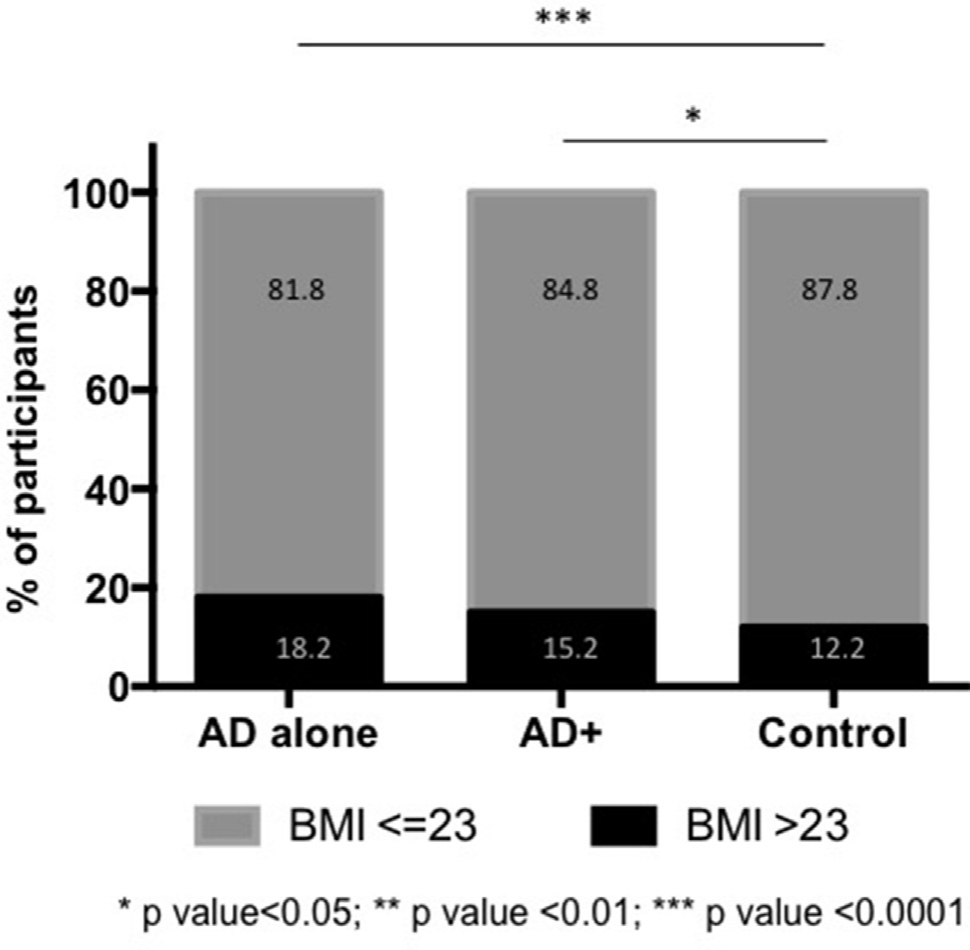
Characterization by Body Mass Index (BMI) BMI ≤ 23 was used as the reference category; *p < 0.05; **p < 0.01; ***p < 0.0001.

For this analysis, a cut-off value of BMI > 23 was used to define overweight individuals.^26^ Participants with AD were shown to be associated with a higher BMI compared to the Control group. The difference was more pronounced in the AD alone group (Odds Ratio [OR]: 1.593; 95% Confidence Interval [CI]: 1.21–2.08; p < 0.001) compared to the AD+ group (OR: 1.27; 95% CI: 1.02–1.59; p < 0.05) (Fig. 2). Stratification by gender showed that the difference was observed mostly in males, unlike in females in whom this difference was inversed.

### Multiple regression analysis

A multiple regression analysis was performed to assess the influence of an increased BMI on the AD alone and AD+ groups compared to the Control group. The analysis showed that a higher BMI correlated with an increased risk in both the AD alone and AD+ groups compared to the Control group. Multivariate analysis with other confounding factors, such as the number of years residing in Singapore, household income, sensitization, and gender showed that a high BMI (>23) was associated with the presentation of AD alone (OR: 2.09; 95% CI: 1.27–3.44; p < 0.01). However, this association was not observed for the presentation of AD+. Similarly, when controlled for other confounding factors (number of years in Singapore, household income, gender, and BMI), sensitization (skin prick positive responses to HDM) was highly associated with the presentation of AD+ (OR: 3.79; 95% CI: 3.03–4.74; p < 0.0001) but not associated with AD alone (OR: 0.86; 95% CI: 0.69–1.05; p > 0.05).

## Discussion

The aim of this cross-sectional, epidemiological study was to characterize the phenotypic differences between AD groups and identify factors associated with the different presentations of AD.

The results showed that sensitization was not an associated factor for participants in the AD alone group, in contrast to those in the AD+ group. Based on these findings, it may be inferred that the disease pathophysiology is different between participants in the AD alone group and those in the AD+ group. Further investigation is warranted to confirm this inference. Previous studies showed that children with an early onset of AD have an increased risk of developing asthma or AR.^18^, ^21^, ^27^, ^28^, ^29^ Though the age of onset was assessed during the study, since it was a cross sectional study, the ascertainment may not have been very accurate. The data was thus not evaluated.

The study discussed herein was the first to identify the role of BMI in AD alone. Previous studies involving US, European, and Asian populations have demonstrated a link between increased BMI and atopic diseases (asthma).^11^, ^30^, ^31^ Classification according to BMI (BMI ≤ 23 used as reference) revealed a significant difference in the AD alone and AD+ groups compared to the Control group. Numerous studies conducted in Asia, Europe, and North America have also reported an association between AD and obesity.^31^, ^32^, ^33^, ^34^, ^35^, ^36^, ^37^, ^38^, ^39^ However, this association was not observed in subjects with symptoms of dermatitis and an absence of atopy. This further strengthens the need to stratify study populations. In the population discussed herein, an increased BMI was reported in the AD alone group, with the difference being greater than in the AD+ group. This finding is suggestive of an association between AD alone and high BMI.

A multiple regression analysis was performed to adjust the OR for BMI using various parameters such as years of residence in Singapore, income, gender, and atopy. For the AD alone group, the association with BMI was more pronounced, and a higher BMI was linked to an even higher risk of disease in the absence of the above confounding factors. However, no significant difference was observed in the AD+ group after adjustment. Although there was no major difference in the food habits or physical activity between the groups, BMI was a factor associated with the disease. On adjusting for confounding factors such as gender, years of residence in Singapore, and household income, BMI was still found to be associated with AD in the AD alone group. Therefore, the patient's BMI should be taken into consideration when studying AD.

The investigators acknowledge the following shortcomings of this study. Firstly, the study population consisted exclusively of young university students with a mean age of 22.1 years and persistent syndrome which is not representative of the overall population in Singapore. However, similar to a study conducted in 2002, it is helpful in studying AD prevalence and risk factors in a section of the Singapore population.^43^ Nevertheless, studies have reported a higher prevalence of AD adults and adult onset of AD in Asians.^40^, ^41^, ^42^ Secondly, the classification of AD was based on self (participant)-reported symptoms. Also, a majority of the study participants are those who suffer from a mild form of AD and hence the results might not be applicable to more severe forms of the disease. This is similar to the mild moderate sufferers seen in a larger cohort study conducted in school going children in Singapore.^43^ Lastly, the collection of data regarding the prescription of medications was also based on participant-reported information.

## Conclusions

This study showed that the two presentations of AD (AD alone and AD with other allergic diseases) may have different underlying pathogenesis and associated risk factors.

## Declarations

### Ethics approval and consent to participate

This study was conducted in accordance with the principles of the Declaration of Helsinki and Good Clinical Practices, and in compliance with local regulatory requirements. It was approved by an institutional review board of the National University of Singapore (IRB reference numbers: NUS07-023, NUS10-373 and NUS13-075), and all subjects provided written informed consent prior to study procedures.

### Consent for publication

Not applicable.

### Availability of data and material

The datasets used and/or analyzed during the current study are available from the corresponding author on reasonable request.

## Conflicts of interest

CFT has received research support from the Singapore Ministry of Education Academic Research Fund, the Singapore Immunology Network, the National Medical Research Council (NMRC; Singapore), and the Agency for Science Technology and Research (A*STAR; Singapore; N-154-000-038-001, R-154-000-404-112, R-154-000-553-112, R-154-000-565-112, R-154-000-630-112, R-154-000-A08-592, R-154-000-A27-597, SIgN-06-006, SIgN-08-020, and NMRC/1150/2008); has received consultancy fees from the Sime Darby Technology Center, Olam International and First Resources Ltd; and is employed by the National University of Singapore. MSA, SYY, SBK, AAK, and AR have no conflicts of interest to declare. BRS, PC, and BV are employees of Nestlé Skin Health–Galderma R&D.

## Authors' contributions

CFT, BRS, PC and BV planned and conceptualized the study; MSA, SYY, SBK, AAK, AR and CFT performed the epidemiological and laboratory studies, MSA, BRS, SYY, BV, PC and CFT analyzed and interpreted the data. CFT, MSA, BRS, PC and BV contributed in writing the manuscript. All authors read and approved the final manuscript.

## Funding

Nestlé Skin Health–Galderma R&D (CUTIS team) was the sponsor of this study. The sponsor was not implicated in data collection. The sponsor participated in the elaboration of the study design, and the analysis and interpretation of the results. The sponsor was fully involved in the writing and the decision to submit this article for publication.
