# Supplementary material for: Different phenotypes and factors associated with atopic dermatitis in the young adult Singaporean Chinese population: A cross-sectional study
Source: World Allergy Organ J. 2019 Jan 26;12(1):100008. doi: 10.1016/j.waojou.2018.11.006 (PMC6439406; doi:10.1016/j.waojou.2018.11.006)
Supplement: Multimedia component 1 [file mmc1.doc]

**Supplementary Table 1.** AD alone represents atopic dermatitis without asthma or allergic rhinitis. AD with allergic diseases represents AD with asthma, allergic rhinitis, or both. Values are expressed at mean± standard deviation or number (percentage %).

|  | **AD alone group**  **(N=496)** | **AD with allergic diseases group**  **(N=894)** | **Control group**  **(N=3214)** |
| --- | --- | --- | --- |
| **Physical activity** |  | | |
| 1-2 times per week | 265 (54%) | 492 (55%) | 1775 (56%) |
| Everyday | 45 (9%) | 114 (13%) | 356 (11%) |
| Occasionally | 183 (37%) | 288 (32%) | 1062 (33%) |
| **Smoking** |  | | |
| Smoker | 6 (1.3%) | 8 (1%) | 23 (.7%) |
| Ex-smoker | 4 (0.8%) | 17 (2.1%) | 33 (1.1%) |
| Non-smoker | 442 (97.9%) | 784 (96.9%) | 2854 (98.2%) |
| **Positive response for prescription of medications** |  | | |
| Anti-histamines | 52 (20%) **** | 219 (38%) ^††††^ | 123 (9%) |
| Bronchodilators | 5 (2%) | 120 (21%) ^††††^ | 14 (1%) |
| Inhaled steroids | 0 | 84 (14.5%) ^††††^ | 4 (0.05%) |
| Oral steroids | 15 (6%) | 64 (11%) ^††††^ | 25 (2%) |
| Nasal spray | 19 (7%) | 177 (32%) ^††††^ | 74 (5%) |
| Topical steroids | 50 (12.5%) **** | 171(31%) ^††††^ | 65 (5%) |
| Antibiotics | 53 (13%) **** | 88 (17%) ^††††^ | 68 (5%) |
| Moisturizers | 59 (15%) **** | 149 (29%) ^††††^ | 82 (6%) |

*p<0.05, **p<0.01 and ***p<0.001 and ****p<0.0001 (AD alone vs. Control)

^†^p<0.05, ^††^p<0.01, ^†††^p<0.001 and ^††††^p<0.0001 (AD+ vs. Control)

**Supplementary Table 2.** AD alone represents atopic dermatitis without asthma or allergic rhinitis. AD with allergic diseases represents AD with asthma, allergic rhinitis, or both. Values are expressed at mean± standard deviation or number (percentage %).

|  | **AD alone group**  **(N=496)** | **AD with allergic diseases group**  **(N=894)** | **Control group**  **(N=3214)** |
| --- | --- | --- | --- |
| **Meat** |  | | |
| Occasionally | 14 (3%) | 23 (2.5%) | 52 (1.5%) |
| 1-2 per week | 54 (11%) | 87 (10%) | 323 (10%) |
| Everyday | 427 (86%) | 780 (87.5%) | 2825 (88.5%) |
| **Seafood** |  | | |
| Occasionally | 46 (9%) | 63 (7%) | 196 (6%) |
| 1-2 per week | 222 (45%) | 409 (46%) | 1523 (48%) |
| Everyday | 225 (46%) | 419 (47%) | 1477 (46%) |
| **Fruits** |  | | |
| Occasionally | 15 (3%) | 40 (4%) | 102 (3%) |
| 1-2 per week | 163 (33%) | 281 (31.5%) | 1027 (32%) |
| Everyday | 317 (64%) | 571 (64.5%) | 2067 (65%) |
| **Vegetables** |  | | |
| Occasionally | 13 (3%) | 33 (3.6%) | 62 (2%) |
| 1-2 per week | 64 (13%) | 100 (11%) | 376 (12%) |
| Everyday | 416 (84%) | 760 (85.4%) | 2747 (86%) |
| **Pulses (eg: lentils, beans, peas)** |  | | |
| Occasionally | 93 (19%) | 226 (25%) ^††^ | 633 (20%) |
| 1-2 per week | 296 (60%) | 488 (55%) | 1971 (62%) |
| Everyday | 103 (21%) | 174 (20%) | 570 (18%) |
| **Cereals** |  | | |
| Occasionally | 38 (7.7%) | 78 (9%) | 305 (9.5%) |
| 1-2 per week | 206 (42%) | 364 (41%) | 1217 (38%) |
| Everyday | 247 (51.8%) | 448 (50%) | 1654 (52.5%) |
| **Pasta** |  | | |
| Occasionally | 189 (4%) | 324 (36.5%) | 1258 (40%) |
| 1-2 per week | 250 (51%) | 468 (52.5%) | 1592 (50%) |
| Everyday | 54 (45%) | 99 (11%) | 334 (10%) |
| **Rice** |  | | |
| Occasionally | 12 (2%) | 11 (1%) | 68 (2%) |
| 1-2 per week | 52 (11%) | 92 (10%) | 312 (10%) |
| Everyday | 429 (87%) | 784 (89%) | 2809 (88%) |
| **Butter** |  | | |
| Occasionally | 193 (4%) ^*^ | 368 (42%) | 1467 (46%) |
| 1-2 per week | 240 (49%) | 416 (47%) | 1353 (42.5%) |
| Everyday | 59 (47%) | 103 (11%) | 362 (11.5%) |
| **Margarine** |  | | |
| Occasionally | 248 (51%) | 488 (55%) | 1646 (52%) |
| 1-2 per week | 199 (41%) | 321 (36%) | 1232 (39%) |
| Everyday | 43 (8%) | 79 (9%) | 292 (9%) |
| **Nuts** |  | | |
| Occasionally | 193 (39%) | 395 (44%) | 1386 (44%) |
| 1-2 per week | 250 (51%) | 413 (46%) | 1571 (49%) |
| Everyday | 49 (10%) | 82 (9%) | 237 (7%) |
| **Potato** |  | | |
| Occasionally | 87 (18%) | 169 (19%) | 525 (16%) |
| 1-2 per week | 327 (67%) | 585 (66%) | 2196 (69%) |
| Everyday | 77 (15%) | 137 (15%) | 468 (15%) |
| **Milk** |  | | |
| Occasionally | 95 (19%) | 188 (21%) | 625 (20%) |
| 1-2 per week | 237 (48%) | 400 (45%) | 1509 (47%) |
| Everyday | 160 (33%) | 303 (34%) | 1053 (33%) |
| **Eggs** |  | | |
| Occasionally | 11 (2%) | 33 (4%) | 112 (3.5%) |
| 1-2 per week | 208 (42%) | 371 (42%) | 1463 (46%) |
| Everyday | 273 (56%) | 486 (54%) | 1608 (50.5%) |
| **Fast food** |  | | |
| Occasionally | 184 (37.5%) | 335 (38%) | 1205 (38%) |
| 1-2 per week | 270 (55%) | 487 (55%) | 1775 (56%) |
| Everyday | 36 (7.5%) | 66 (7.5%) | 202 (6%) |
| **Yoghurt** |  | | |
| Occasionally | 177 (9%) | 232 (12%) | 83 (12%) |
| 1-2 per week | 367 (19%) | 361 (18%) | 159 (23%) |
| Everyday | 1359 (72%) | 1381 (70%) | 440 (65%) |

*p<0.05, **p<0.01 and ***p<0.001 and ****p<0.0001 (AD alone vs. Control)

^†^p<0.05, ^††^p<0.01, ^†††^p<0.001 and ^††††^p<0.0001 (AD+ vs. Control)
